# Differences in Technical Aspects of Primary Sleeve Gastrectomy Prior to Redo Bariatric Surgery—A Multicenter Cohort Study (PROSS Study)

**DOI:** 10.3390/medicina59040799

**Published:** 2023-04-20

**Authors:** Piotr Zarzycki, Justyna Rymarowicz, Piotr Małczak, Magdalena Pisarska-Adamczyk, Rafał Mulek, Artur Binda, Natalia Dowgiałło-Gornowicz, Piotr Major, PROSS Collaborative Study Group

**Affiliations:** 1 Department of Medical Education, Jagiellonian University Medical College, 30-688 Krakow, Poland; piotr.zarzycki@uj.edu.pl (P.Z.); magdalenapisarska@interia.pl (M.P.-A.); 2 2nd Department of General Surgery, Jagiellonian University Medical College, 30-688 Krakow, Poland; justyna.rymarowicz88@gmail.com (J.R.);; 3 EuroMediCare Specialist Hospital and Clinic, 54-144 Wroclaw, Poland; rachfal.rm@gmail.com; 4 Department of General, Oncological and Digestive Tract Surgery, Centre of Postgraduate Medical Education, Orłowski Hospital, 00-416 Warsaw, Poland; artur.binda@interia.com; 5 Department of General, Minimally Invasive and Elderly Surgery, Collegium Medicum, University of Warmia and Mazury, 10-045 Olsztyn, Poland; natalia.dowgiallo@gmail.com

**Keywords:** bariatric surgery, revisional obesity surgery, primary bariatric procedure, technical aspect, insufficient weight loss, weight regain

## Abstract

*Background and Objectives:* Although the technical simplicity of laparoscopic sleeve gastrectomy is relatively well understood, many parts of the procedure differ according to bariatric surgeons. These technical variations may impact postoperative weight loss or the treatment of comorbidities and lead to qualification for redo procedures. *Materials and Methods*: A multicenter, observational, retrospective study was conducted among patients undergoing revision procedures. Patients were divided into three groups based on the indications for revisional surgery (insufficient weight loss or obesity-related comorbidities treatment, weight regain and development of complications). *Results*: The median bougie size was 36 (32–40) with significant difference (*p* = 0.04). In 246 (51.57%) patients, the resection part of sleeve gastrectomy was started 4 cm from the pylorus without significant difference (*p* = 0.065). The number of stapler cartridges used during the SG procedure was six staplers in group 3 (*p* = 0.529). The number of procedures in which the staple line was reinforced was the highest in group 1 (29.63%) with a significant difference (0.002). Cruroplasty was performed in 13 patients (*p* = 0.549). *Conclusions*: There were no differences between indications to redo surgery in terms of primary surgery parameters such as the number of staplers used or the length from the pylorus to begin resection. The bougie size was smaller in the group of patients with weight regain. Patients who had revision for insufficient weight loss were significantly more likely to have had their staple line oversewn. A potential cause could be a difference in the size of the removed portion of the stomach, but it is difficult to draw unequivocal conclusions within the limitations of our study.

## 1. Introduction

Since restrictive and metabolic surgery was first performed for severe obesity, the number of bariatric procedures has been steadily increasing annually, according to ASMBS statements [1]. The most common procedure worldwide is laparoscopic sleeve gastrectomy [2]. Although the metabolic and weight loss effects of bariatric procedures are well known, their durability varies in some patients [3]. Insufficient weight loss after primary treatment, weight regain, reflux disease (GERD), and incomplete diabetes or comorbidities remission are all indications for bariatric surgery [4,5]. The sleeve gastrectomy procedure still has no established gold standard. Although the technical simplicity of laparoscopic sleeve gastrectomy is relatively well understood, many parts of the procedure differ according to bariatric surgeons, such as the size of the calibrating tube, the number of stapler firings, or oversewing of the staple line, omentopexy, omentectomy, or the addition of a band around the upper part of the sleeve (“Banded Sleeve Gastrectomy”) [6,7,8]. These differences can affect the short- and long-term postoperative outcomes and may affect the duration of the procedure, length of hospital stay (LOS), postoperative complications, and also the metabolic results [9]. Various post-operative complications can lead to the necessity for redo surgery. Therefore, we selected a group of patients who required revisional bariatric surgery after primary SG, divided into three groups based on the indications for redo procedure, and assessed the possible relationship between the technique of the primary surgery and the necessity for revision treatment.

## 2. Materials and Methods

### 2.1. Study Design

A multicenter, retrospective cohort study was conducted. The data were collected between 2019 and 2020 from participating Polish Bariatric Centers using the internet-based database. The project was supported by the Metabolic and Bariatric Surgery Chapter and Videosurgery Chapter of the Association of Polish Surgeons. The study group named Poland Revisional Obesity Surgery Study (PROSS) was created. In each center, surgeons, nurses, and bariatric care coordinators were involved in collecting data concerning bariatric patients undergoing revisional surgery to create a comprehensive database. The inclusion criterion was undergoing revisional bariatric treatment. The Strengthening the Reporting of Observational Studies in Epidemiology (STROBE) statement was used in the design of the study and to prepare the manuscript [10].

The baseline demographic characteristics included: sex, age, maximal weight, weight before primary surgery, weight before revisional surgery, height, Body Mass Index (BMI), diabetes mellitus type 2 (T2D), hypertension, duration of obesity, treatment with non-steroidal anti-inflammatory drugs (NSAIDs), tobacco smoking, and alcohol consumption. Additionally, they included data concerning primary surgery (prior intragastric balloon placement, type of surgery, LOS, incidence of complications), outcomes of primary bariatric treatment (lowest body weight, T2D remission, hypertension remission, technical aspects of primary LSG—the size of calibrating bougie, length from the pylorus to the first stapler, number of stapler firings, staple line oversuturing, cruroplasty), data concerning revisional surgery (indication for revisional surgery, LOS, type of surgery, complications), and outcomes of the redo procedure (weight reduction, T2D remission, hypertension remission).

The database consists of 799 patients who underwent revisional bariatric surgery from May 2005 to January 2020. A total of 477 (59.7%) patients who underwent laparoscopic sleeve gastrectomy as a primary bariatric procedure were included in the analysis. Patients were divided into three groups based on the indications for revisional surgery: insufficient weight loss or the unsatisfactory resolution of obesity-related comorbidities (group 1), weight regain (group 2), and development of complications, such as GERD (group 3). If a patient had more than one indication for redo bariatric surgery, they were classified into the appropriate group based on the main indication. 

Weight regain was defined as achieving over 50% EWL (excess weight loss) at postoperative period, and after that as weight gain to BMI > 40 kg/m^2^ or BMI > 35 kg/m^2^ with obesity-relative comorbidities. Insufficient weight loss was defined as not achieving over 50% EWL at postoperative period. Insufficient comorbidities treatment was defined as not obtaining complete remission of hypertension and/or diabetes after RBS. Group 3 consisted of patients who developed complications after primary bariatric procedure and required redo surgery.

**Figure 1:**
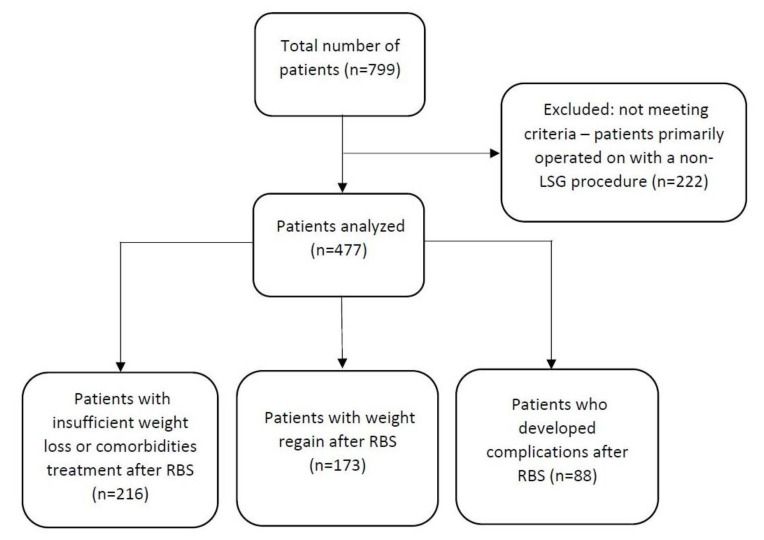
Flowchart of patients in the study.

A flowchart of the patients included in the study is presented in Figure 1.

### 2.2. Surgical Technique and Perioperative Care

The perioperative care protocols, including the preoperative, intraoperative, and postoperative interventions, were standard for each center participating in the study. The surgical technique was mostly standard for each center participating in the study, but there were a few modifications, such as the size of the calibrating tube, the distance from the pylorus to the first stapler, the number of stapler firings, oversuturing of the staple line, and cruroplasty. The preoperative workup, perioperative care, and follow-up visits were coordinated by the multidisciplinary bariatric team in each center, including surgeons, physicians, nurses, dieticians, and psychologists. 

### 2.3. Statistical Analysis

We conducted a descriptive statistical analysis. All data were analyzed using Statistica version 13.1PL (StatSoft Inc., Tulsa, OK, USA). Number and percentage were used for categorical variables. Mean and standard deviation was used for continuous variables with normal distribution. Median and range were used for non-normally distributed data. The normality of distribution for quantitative variables was checked by the Shapiro–Wilk test. The Kruskal–Wallis test was used for skewed data. Results were considered statistically significant when the *p*-value was found to be less than 0.05.

### 2.4. Ethical Considerations

The data were completely anonymized, and no patient personal details or hospital information were collected in the database. All procedures were performed in accordance with the ethical standards of the 1964 Declaration of Helsinki and its later amendments (Fortaleza). The protocol has been registered at https://clinicaltrials.gov/ (accessed on 5 November 2021) (NCT05108532). This study did not implement any changes in the surgical treatment and perioperative care protocols. The course of the study was closely monitored by the primary investigator, who processed and verified any missing or unclear data submitted to the central database. The study was approved by the Bioethics Committee of the Regional Chamber of Physicians, District of Warmia and Mazury, Poland (23/2021/VIII).

## 3. Results

### 3.1. Group Characteristics

The group of patients who underwent revisional bariatric surgery after primary laparoscopic sleeve gastrectomy consisted of 477 patients, 264 (76.31%) women and 113 (23.69%) men. The mean age was 40.37 ± 10.15 kg/m^2^. The mean BMI before primary sleeve gastrectomy was 46.99 ± 9.83 kg/m^2^. The lowest BMI after primary surgery was 34.34 ± 7.84 kg/m^2^. The mean BMI before secondary (revision) surgery was 39.85 ± 8.29 kg/m^2^. After revisional surgery, the mean BMI was 30.47 ± 10.79 kg/m^2^. Type 2 diabetes (T2D) was diagnosed in 132 patients (27.67%) prior to bariatric treatment. A total of 217 (45.49%) patients were diagnosed with preoperative hypertension. The mean LOS was 3.45 days (2–25) after the primary sleeve gastrectomy and 3.32 days (1–36) after the revisional surgery. The basic characteristics of the groups are shown in Table 1. 

### 3.2. Technical Aspects of Primary Sleeve Gastrectomy

The median bougie size was 36 (32–40) (*p* < 0.05). In 246 (51.57%) patients, the resection part of sleeve gastrectomy started 4 cm from the pylorus. In 121 (25.37%) patients the first stapler was placed 6 cm from the pylorus (*p* = 0.065). The median number of stapler firings used in primary SG was five (three to nine) (*p* = 0.529). In 106 patients (22.22%), there was oversuturing of the staple line after sleeve resections (*p* < 0.05). Cruroplasty was performed in thirteen patients (2.73%), and two of them (15.4%) underwent revisional surgery for intractable GERD symptoms (*p* = 0.549). The differences in the surgical technique during the primary sleeve gastrectomy, divided into groups in terms of indications for revision surgery, are shown in Table 2.

### 3.3. Revision Bariatric Procedures

OAGB was the most common type of surgery used as revisional bariatric surgery after primary sleeve gastrectomy—270 cases (56.6%). RYGB was the second most common choice after primary SG. ReSG, SADI-S, SASI, or BPD-DS were rarely used as revision bariatric surgery after SG. Types of revisional bariatric surgery performed after primary SG are presented in Table 3.

## 4. Discussion

Laparoscopic sleeve gastrectomy is the most commonly performed bariatric surgery in Poland. Data gathered show that in the majority, SG is also the primary procedure before revision. The primary indication for revision surgery after SG was insufficient weight loss or comorbidities treatment (45.28%). In this group, BMI following primary surgery was the highest compared to other groups, and the prevalence of comorbidities (hypertension and diabetes) was high as well. Patients requiring revision surgery due to complications (group 3) had the lowest BMI among all patients, regardless of the time at which it was measured (before primary SG or before and after revision surgery). 

Since the first sleeve gastrectomy was performed, the surgical technique has been gradually developing over time [11]. Despite the widespread opinion that SG is a well-standardized technique, our study confirms that there are several possible modifications of this procedure. The particular steps vary depending on the surgical center, the preferences of the surgeon performing the procedure, or the available surgical equipment. Modifications of the procedure may change the prevalence of complications, and also affect postoperative weight loss and prevent obesity recurrence.

### 4.1. Technical Aspects of Using a Calibrating Bougie

In our study, the 36 Fr probe was the most frequently used size. The median size of the probe was slightly smaller (35 Fr) in the group of patients with weight regain, and the results were statistically significant. Due to the different sizes of calibration bougies, numerous analyses of postoperative results were carried out. The available bougie sizes ranged from 28 to 50 Fr, but the most commonly used diameters were from 34 to 38 Fr, which is consistent with the results of our study. Po-Chih Chang et al., in a network meta-analysis, showed that intraoperative calibration with a bougie sized 33–36 Fr was an optimal choice to balance the effectiveness and safety for patients with morbid obesity undergoing SG [12]. On the other hand, calibration with a tube greater than 40 Fr was associated with lower weight loss in the postoperative period. Nevertheless, calibration with a tube smaller than 36 Fr does not lead to better weight loss after surgery, but significantly increases the risk of leakage and clinical symptoms of stenosis. Failure to use a calibration tube may lead to the lack of adequate gastric resection and subsequent inadequate weight loss in the postoperative period [13]. The results of the Bougie Sleeve Trial (BOUST), which is a multicentre single-blinded randomized trial, may show whether the use of a larger calibration bougie during SG is associated with lower postoperative gastric leak occurrences without impairing mid-term weight loss and quality of life. In this trial, participants will be randomized into two groups: SG performed using a 48-Fr diameter calibration bougie, and SG performed by a standard (34 to 38-Fr diameter) calibration bougie [14]. In conclusion, a larger diameter of calibrating bougie does not have a clear relationship with worse weight loss and the subsequent need for revision for this reason, while a smaller calibrating bougie may cause more complications in the form of stenoses, which in turn may require revision surgery. M E Abd Ellatif et al. suggest that a smaller bougie size was associated with significant %EWL [15]. Due to the achievement of significant weight loss in patients after primary SG with the use of a smaller calibration bougie, the indication for a potential revision procedure will be weight regain or the development of complications. It could be an explanation for the potential reason for the statistically smaller calibrating probe diameter in the group of patients undergoing revision surgery due to weight regain.

### 4.2. Technical Aspects of the Distance from the Pylorus of the First Stapler

In our study, 4 cm was the most frequently chosen distance from the pylorus in all groups as the start of resection. However, in group 3, both the smallest (2 cm) and the greatest (>6 cm) distance from the pylorus was chosen more often compared to the other groups. This might have an impact on the postoperative outcomes, bearing in mind that this is a group of patients in which the indication for revision surgery was the occurrence of complications. Currently, the available literature provides heterogeneous research results. Hassan et al., in their study, concluded that the distance of the first incision from the pylorus had no impact on the percentage of excessive weight loss, the resolution of comorbidities, changes in the quality of life, or the occurrence of complications [16]. Similar conclusions were presented in the study comparing distances of 2 cm and 6 cm from the pylorus, in which it was shown that both methods resulted in a significant loss of body weight after surgery, and comorbidities were alleviated with a slight predominance of the 6 cm group [17]. However, another analysis suggested that distances less than 3 cm from the pylorus were associated with better and sustainable weight loss, but appeared to cause more nausea and vomiting in the early postoperative period [18]. Antral resection with staples starting 2 cm from the pylorus is expected to provide a more restrictive SG effect and achieve greater weight loss. Initiating the resection 6 cm from the pylorus leaves the antrum of the stomach with the intention of maintaining its contractile force, thus improving gastric emptying, reducing intragastric pressure and consequently reducing the risk of leakage. The decision about resection distance from the pylorus is mainly based on choosing between the two options above. In summary, beginning sleeve resection at a distance of less than 3 cm may be associated with better and more sustained weight loss, but at the same time may be associated with increased complications and therefore the need for revision surgery. The results of our study are consistent with the above conclusions.

### 4.3. Technical Aspects of the Number of Stapler Firings

In our data, the highest median number of stapler cartridges used during the SG procedure was six staplers, in group 3, but the differences were not statistically significant. Major et al., in their study, showed that the absolute number of stapler firings was significantly related to a higher rate of postoperative complications, which is comparable to our data [19]. 

### 4.4. Technical Aspects of the Oversuturing the Staple Line

The number of procedures in which the staple line was reinforced was the highest in group 1 (29.63%), with statistical significance. The reinforcement of the staple line is a constant topic of debate regarding its effect on postoperative outcomes. Aiolfi et al., in their systematic review and network meta-analysis of randomized controlled trials, compared the different types of staple line reinforcement (SLR). The result of this study showed that SLR compared to non-reinforcement may be associated with a reduced risk of postoperative bleeding, staple line leakage, and overall complications in spite of a longer operative time [20]. Possible methods of reinforcement consist of suture oversewing, glue reinforcement, bioabsorbable staple line reinforcement (Gore^®^ Seamguard^®^), and clips reinforcement. In our study, there is no information on whether a different staple line reinforcement (except oversewing) was performed. According to recent studies, suture oversewing decreased the risk of postoperative bleeding and may lead to fewer staple-line complications [21,22]. Despite the fact that different methods of SLR have been developed, staple line leakage still occurs. Omentopexy may be a feasible procedure for decreasing morbidity and gastric leak rate. Even though the results are promising, the procedure needs to be researched more in randomized controlled studies to draw solid conclusions [23]. Consistent with the above data, staple line reinforcement could reduce bleeding or reoperation rates following SG and the potential need for revision surgery due to complications. In our study, more patients with staple oversewing during SG required revision surgery due to insufficient weight loss or comorbidity treatment. A potential cause could be a difference in the size of the removed portion of the stomach. In the case of later suturing of the staple line, the surgeon performs a "smaller" resection, leaving a wider gastric tube for later, safer reinforcement of the staple line without the risk of narrowing the remaining part of the stomach. When SG is performed without suturing the staple line, the surgeon can perform a "more aggressive" resection by removing most of the stomach along with the ghrelin and leptin receptors it contains. Leaving most of the stomach may result in an inadequate response to the primary SG and the need for revision procedures. This hypothesis has not been confirmed in the available literature and requires additional research.

### 4.5. Technical Aspects of the Cruroplasty

A lot of patients who qualified for bariatric surgery complain of symptoms of gastroesophageal reflux. A strong correlation between BMI and GERD was described and high BMI (such as in obesity or overweight patients) is an independent risk factor for GERD symptoms and esophageal erosions [24]. In our data, 13 patients had cruroplasty during SG and two patients (15.4%) required revisional surgery due to the recurrence of gastroesophageal reflux (one patient had only GERD alone, but the second patient had obesity recurrence with GERD symptoms). In a randomized control study, Masoud Sayadi Shahraki et al., showed that the addition of cruroplasty to sleeve gastrectomy to minimize de novo GERD symptoms after the surgery lacks effectiveness and is not recommended [25]. On the other hand, in their retrospective cohort study, Alaa Abbas Sabry et al. described how hiatal hernia repair during a laparoscopic sleeve gastrectomy procedure could eliminate GERD symptoms in morbid obesity patients with pre-operative hiatal hernia and GERD [26]. In a study comparing sleeve gastrectomy with cruroplasty alone to SG with cruroplasty reinforced with bioabsorbable mesh, 18.4% of patients with cruroplasty had intrathoracic migration after 5 years of follow-up. Authors suggested that sleeve gastrectomy combined with posterior cruroplasty (simple or reinforced with mesh) could be feasible and effective in selected patients, but overall hiatal hernia recurrence after 5 years follow-up was up to 10.7% [27]. In a recent meta-analysis, Małczak et al. showed no differences in the GERD rate between hiatal hernia repair during sleeve gastrectomy in comparison to sleeve gastrectomy alone [28]. In another meta-analysis, authors showed that sleeve gastrectomy with hiatal hernia repair (HHR) had a better outcome for GERD remission, esophagitis reduction, and GERD-HRQL improvement, but no influence on de novo GERD rates compared to sleeve gastrectomy alone [29]. Concomitant cruroplasty with laparoscopic sleeve gastrectomy is still a controversial procedure for eliminating GERD symptoms. RYGB could be a better option for patients with obesity and pre-operatively diagnosed advanced gastroesophageal acid reflux disease. 

### 4.6. Revisional Bariatric Surgery after Primary Sleeve Gastrectomy

After the primary sleeve gastrectomy surgery, several possible revision procedures are Re-SG (“Repeated Sleeve Gastrectomy”), SADI-S (“Single Anastomosis Duodeno–Ileal bypass with Sleeve Gastrectomy”), OAGB (“One Anastomosis Gastric Bypass”), RYGB (“Roux-en-Y Gastric Bypass”), or BPD-DS (“BilioPancreatic Diversion with Duodenal Switch”). According to the data available in the literature, RYGB has been the gold standard as the revisional procedure after SG [30]. In our study, OAGB was the most frequently performed revision procedure (majority of patients group 1 and 2), while the second most common was RYGB (majority of patients in group 3). The novel, more advanced surgeries (such as SADI-S or SASI), where the long-term results are still under investigation, were performed in patients who experienced obesity recurrence or inadequate weight loss after the primary surgery [31]. In the group of patients whose indication for revision was the development of complications, the procedures with the most known postoperative effects were used. New treatments were not used in this group, most likely to avoid the occurrence of further complications possible after experimental procedures. However, there are no clear guidelines about the most appropriate method after sleeve gastrectomy and the factors affecting the selection of the best method.

### 4.7. Limitations

**Figure 2:**
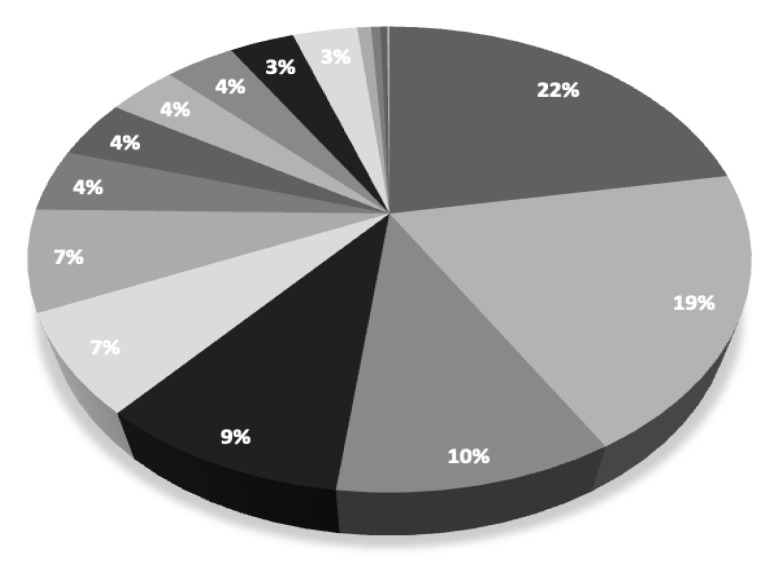
**Percentage of the number of records entered into the database by bariatric centers. Legend:** Each color on the chart corresponds to a different bariatric center which entered records into the database. The numbers on the chart indicate the percentage contribution of the number of records entered by the bariatric center compared to all records in the database.

This study has a lot of limitations. A retrospective study involves only the analysis of the obtained data, and has lack of possible randomization and no possibility of interfering with the patient’s treatment process. A randomized study would provide more reliable results. Assignment to the group related to the indications for bariatric revision surgery was based on the patients’ main symptoms. In some cases, patients have more than one indication qualifying for revision surgery (e.g., weight regain and GERD symptoms). A center introducing a much larger number of patients may affect the statistical results and distort the subsequent analysis. The operating technique of the center with the highest number of patients entering the base may negatively affect the analysis of the population of all patients in the base. Three bariatric centers entered into the database more than 50 percent of records. The percentage of the number of records entered into the database by bariatric centers is shown in Figure 2. 

## 5. Conclusions

Despite its widespread use, sleeve gastrectomy still shows differences in surgical techniques used. There were no differences between indications to redo surgery in terms of primary surgery parameters such as the number of staplers firing, the distance from the pylorus to begin resection, or cruroplasty. The statistical difference was in the bougie size, which was smaller in the group of patients with weight regain. There was also a statistical difference in the oversewing of the staple line, which was more common in patients revised for insufficient weight loss. We hypothesize that this may be caused by the difference in the size of the removed portion of the stomach, but it is difficult to draw unequivocal conclusions as a result of the low quality of the data available for analysis. Due to the limitations of the selected group and retrospective study, further, randomized, studies are required to more accurately assess the impact of changes in the surgical technique of sleeve gastrectomy on long-term results. The study population was a selected group of patients following revision bariatric procedures. Designing a study comparing the obtained results with the group of patients after sleeve gastrectomy without subsequent revision surgery may show interesting results.

## Figures and Tables

**Table 1:** Basic characteristics.

	Group 1 216 (45.28%)	Group 2 173 (36.27%)	Group 3 88 (18.45%)
**Age**	42.06 ± 11.09	38.42 ± 9.62	38.85 ± 10.75
**Sex**			
—women, n (%)	215 (99.54%)	129 (74.57%)	20 (22.73%)
—men, n (%)	1 (0.46%)	44 (25.43%)	68 (77.27%)
**BMI max**, median (range)	50 (44.5–55.7)	48.1 (43.3–53.3)	43.8 (41.5–48)
**BMI before primary SG,** median (range)	46.9 (42.4–52.5)	46.8 (41.9–52.7)	41.8 (38.3–47.3)
**BMI after primary surgery**, median (range)	37.1 (33.5–41.7)	31.8 (28–36.7)	29.4 (17–32.6)
**BMI before secondary (revisional) surgery,** median (range)	39.7 (35.1–45.2)	40.9 (36.5–45.4)	33.2 (29.4–36.6)
**LOS primary SG,** mean (SD)	3.12 ± 2.68	3.77 ± 1.5	3.74 ± 2.77
**LOS revisional surgery,** median (min-max)	3.17 (1–36)	3.49 (2–23)	3.40 (2–25)
**T2D**, n (%)	78 (36.11%)	35 (20.23%)	19 (21.59%)
**HTN**, n (%)	119 (55.09%)	66 (38.15%)	32 (36.36%)
**Post-SG GERD**, n (%)	16 (7.14%)	10 (5.78%)	72 (81.82%)

**Group 1**—insufficient treatment of high weight or comorbidities, **Group 2**—weight regain, **Group 3**—development of complications, such as GERD, **SG**—sleeve gastrectomy, **BMI**—Body Mass Index, **LOS**—length of stay, **T2D**—type 2 diabetes, **HTN**—hypertension, **GERD**—Gastroesophageal Reflux Disease.

**Table 2:** The differences in the elements of the surgical technique during the primary sleeve gastrectomy.

	Group 1	Group 2	Group 3	*p*-Value
**Bougie size**	36 (34–36)	35 (34–36)	36 (34–36)	**0.04**
**Length from the pylorus of the first stapler:**				**0.065**
2 cm	2 (0.99%)	1 (0.58%)	1 (1.14%)	
4 cm	99 (45.83%)	98 (56.65%)	49 (55.68%)	
6 cm	70 (32.41%)	33 (19.08%)	18 (20.45%)	
>6 cm	18 (8.33%)	9 (5.20%)	8 (9.09%)	
no data	27 (12.50%)	32 (18.50%)	12 (13.64%)	
**Number of stapler firings**. mean (median)	5.53 (5)	5.13 (5)	5.55 (6)	**0.529**
**Oversuturing the staple line** n (%):				**0.002**
Yes	64 (29.63%)	28 (16.18%)	14 (15.91%)	
No	152 (70.37%)	145 (83.82%)	74 (84.09%)	
**Cruroplasty** n (%):				**0.549**
Yes	6 (2.78%)	6 (3.47%)	1 (1.14%)	
No	210 (99.22%)	167 (96.53%)	87 (98.86%)	

**Group 1**—insufficient treatment of high weight or comorbidities, **Group 2**—weight regain, **Group 3**—development of complications, such as GERD, **n**—number.

**Table 3:** Types of revisional procedure after primary sleeve gastrectomy.

	All Patients (477)	Group A (216)	Group B (173)	Group C (88)
**OAGB**	270 (56.6%)	148 (68.52%)	81 (46.82%)	41 (46.59%)
**RYGB**	150 (31.45%)	37 (17.13%)	70 (40.76%)	43 (48.86%)
**ReSG**	37 (7.76%)	20 (9.26%)	14 (8.09%)	3 (3.41%)
**SADI-S**	8 (1.68%)	6 (2.78%)	2 (1.16%)	0
**SASI**	7 (1.47%)	3 (1.39%)	4 (2.31%)	0
**other**	5 (1.05%)	2 (0.93%)	2 (1.16%)	1 (1.14%)

**Group 1**—insufficient treatment of high weight or comorbidities, **Group 2**—weight regain, **Group 3**—development of complications, such as GERD, **OAGB**—One Anastomosis Gastric Bypass, **RYGB**—Roux-en-Y Gastric Bypass, **ReSG**—Repeated Sleeve Gastrectomy, **SADI-S**—Single Anastomosis Duodeno–Ileal bypass with Sleeve Gastrectomy, **SASI**—Single Anastomosis Stomach Ileal Bypass.

## Data Availability

Not applicable.
